# How Do We Determine the Efficacy of an Antibacterial Surface? A Review of Standardised Antibacterial Material Testing Methods

**DOI:** 10.3390/antibiotics10091069

**Published:** 2021-09-03

**Authors:** Alexander J. Cunliffe, Peter D. Askew, Ina Stephan, Gillian Iredale, Patrick Cosemans, Lisa M. Simmons, Joanna Verran, James Redfern

**Affiliations:** 1 Department of Natural Sciences, Faculty of Science and Engineering, Manchester Metropolitan University, Chester Street, Manchester M1 5GD, UK; Alexander.J.Cunliffe@stu.mmu.ac.uk; 2 (Industrial Microbiological Services Ltd.) IMSL, Pale Lane, Hartley Whitney, Hants RG27 8DH, UK; Peter.Askew@imsl-uk.com (P.D.A.); Gillian.Iredale@imsl-uk.com (G.I.); 3 (Bundesanstalt für Materialforschung und -prüfung) BAM, Unter den Eichen 87, 12205 Berlin, Germany; Ina.stephan@bam.de; 4 Sirris, Wetenschapspark 3, B-3590 Diepenbeek, Belgium; Patrick.Cosemans@sirris.be; 5 Department of Engineering, Faculty of Science and Engineering, Manchester Metropolitan University, Chester Street, Manchester M1 5GD, UK; L.Simmons@mmu.ac.uk; 6 Department of Life Sciences, Faculty of Science and Engineering, Manchester Metropolitan University, Chester Street, Manchester M1 5GD, UK; J.Verran@mmu.ac.uk

**Keywords:** antimicrobial materials, antimicrobial testing, 22196, antimicrobial surfaces, antibacterial coatings

## Abstract

Materials that confer antimicrobial activity, be that by innate property, leaching of biocides or design features (e.g., non-adhesive materials) continue to gain popularity to combat the increasing and varied threats from microorganisms, e.g., replacing inert surfaces in hospitals with copper. To understand how efficacious these materials are at controlling microorganisms, data is usually collected via a standardised test method. However, standardised test methods vary, and often the characteristics and methodological choices can make it difficult to infer that any perceived antimicrobial activity demonstrated in the laboratory can be confidently assumed to an end-use setting. This review provides a critical analysis of standardised methodology used in academia and industry, and demonstrates how many key methodological choices (e.g., temperature, humidity/moisture, airflow, surface topography) may impact efficacy assessment, highlighting the need to carefully consider intended antimicrobial end-use of any product.

## 1. Introduction

In order for a microorganism to cause disease, it must first reach the potential host. Microorganisms can move around an environment in various but typically passive ways, including via aerosols (inside droplets of water) [1], direct contact between two animated objects [2], and fomites (a contaminated inanimate object) [3]. Some microorganisms can retain their pathogenic potential whilst outside their host for extended periods of time [4], with studies suggesting survival for days and even weeks on inanimate surfaces such as plastics and metals which are often considered to be ‘hygienic surfaces’ [5,6]. Many of these materials are used to construct frequently touched surfaces (FTS) such as door handles, lift buttons, light switches and digital locks [7,8]. Viability of microorganisms on FTS has been demonstrated, and more critically, the evidence of transfer of potentially harmful microorganisms from the FTS to a biological surface (e.g., human skin) via touch has also been reported numerous times in the literature, e.g., [9,10,11]. In the clinical setting, studies highlight bedsteads, supply carts, over-bed tables, lockers, patient bodies, bed-linen, curtains and intravenous pumps as frequently touched when specifically considering interactions between hospital staff and patients [12,13]. These patient-care items can serve as a potential reservoir for pathogenic microorganisms and may be the cause of infection of, and cross-infection between, hospital patients. Non-clinical environmental reservoirs of pathogens can cause further problems where compounding factors also occur, such as on cruise ships where advanced medical treatments are not available [14].

There are various methods that can be utilised to control microorganisms on surfaces in these settings. In clinical environments, methods to chemically disinfect surfaces are often used, but may be performed inadequately, (through poor adherence to cleaning protocols), allowing pathogens to be spread more rapidly throughout wards, following recontamination of disinfected surfaces via contact with fomites [15]. Additionally, disinfectants may themselves drive the evolution of resistance. For example, quaternary ammonium compounds (QAC) have long been considered an effective class of disinfectants and were once thought to be impervious to bacterial resistance. However, an approximate 30% increase of QAC resistance genes has been observed in methicillin-resistant *Staphylococcus aureus* (MRSA) isolates in the years 1990–2010 [16]. To compound this issue further, QAC resistance can undergo horizontal gene transfer to spread resistance and can also propagate the transfer of other antibiotic resistance genes [17]. Disinfecting rooms through exposure to high intensity UV light can be effective at killing the majority of microorganisms it illuminates, but requires all staff and patients to leave the room during the process as a safety requirement [18]. 

Therefore, methods that reduce human error and do not impact on the availability of facilities would be beneficial, such as materials that exhibit antimicrobial activity. Such materials may be innately antimicrobial, produced with a biocide embedded within, or there may be some coating or treatment that confers antimicrobial properties onto the surface—many of which have gained popularity over recent years [19]. For example, in some hospitals and other end-use environments, copper has been exploited as an antimicrobial material (AMM) [20], whilst brass has also been used extensively for FTS, demonstrating an oligodynamic effect [21]. Other materials and additives are now gaining focus in the literature, for example, materials with photocatalytic properties such as certain forms of titanium dioxide, various metal salts and oxides as well as certain dyes [22], other metal ions [23] and some organic agents [24] and materials used in their nano form, such as silver, copper and zinc [25]. Due to the variety and scope of these materials, we will use the term AMM to describe such materials.

The mechanism of action of AMMs can be divided into three major subgroups: active substance release, potentiated surfaces and non-adhesive properties. Each of these approaches has their own advantages and limitations in situ [26]. Contact killing-based claims are the most abundant in the literature, being delivered by both active substance release systems and those with potentiated surfaces. Arguably systems that release less active substance may mitigate any increase in prevalence of antimicrobial resistance associated with AMM’s [27].

Active substance release systems discharge a biocide or antimicrobial agent and this is often triggered through hydration which is then intended to kill microorganisms on the surface and can be highly effective [28]. They can be applied to and incorporated into a wide range of materials (e.g., synthetic polymers) and deployed in a wide range of environments, from FTS in hospital wards to internal devices in patients such as orthopaedic and cardiovascular implants [29], as well as in non-clinical environments such as call buttons and in coatings on hand rails on public transport [30]. 

Potentiated surface-based AMM’s can be fabricated by the inclusion of a biocide, metal, peptides or amines on the surface of the material in order to add an antimicrobial function to that surface [31,32,33]. Common materials used include silver nanoparticles (AgNPs) [25,34,35], copper (Cu) [36,37,38,39], tin disulphide (SnS_2_) [40], ruthenium (Ru) [41,42] and titanium dioxide (TiO_2_) [43]. Within this subcategory of AMMs are photocatalytic materials, that present their antimicrobial effect when exposed to light (e.g., TiO_2_); however, a longer time period is often required to significantly reduce the microbial bioburden, with hours upon exposure to typical solar light [44]. However, doping (e.g., nitrogen [45]) does add further potential for increased efficacy [46]. 

In addition to the mechanisms described above, which focus on a molecule entering or interacting with a cell in some detrimental way, other mechanisms for modifying and potentiating surfaces also exist. For example, carbon nanomaterials (e.g., graphene oxide) have also been shown to be effective at reducing the microbial bioburden on a surface by piercing/damaging cells using jagged, sharp and sturdy surface features [47]. Graphene oxide can also ‘generate’ reactive oxygen species (ROS) [48], and therefore uses multiple features to achieve the desired antimicrobial effect [49]. 

Non-adhesive AMMs have been designed to combat the transfer of microorganisms, as they prevent a microorganism from being able to adhere to the surface, which is therefore also easily cleaned (e.g., by presenting hydrophilic properties [50]). However, it is important to note that an AMM only incorporating this method would likely not reduce the rate of infection in patients, as studies have shown that, particularly bacteria, are able to overcome this ‘line of defence’ when no other antimicrobial properties are being exhibited [51]. 

As described above, there exists a range of applications for which an antimicrobial surface may be a desired option in some settings, with numerous options for manufacturers including different materials/additives and manufacturing processes. However, the variation in approach means that not all AMMs are equal and are likely to all exhibit individual levels of efficacy specific to their product design and intended end-use. As the market for these materials increases, manufacturers of AMMs are required to demonstrate their materials work as intended—meaning efficacy testing is a critical component in AMM development, sale, purchase and end-use decision-making.

## 2. Testing the Efficacy of an AMM

Standardised test methods are a necessary and important step in the development of a novel antimicrobial material. To define a material as antimicrobial, efficacy should be assessed under reproducible conditions that mimic later in-use environments. If the predetermined threshold (usually a 2 or 3-log reduction in viable cells although often individually agreed upon by all parties involved) is not met, then the surface cannot be considered as antimicrobial. This process should enable those interested in AMM’s to ascertain a level of confidence in their material, providing some preliminary positive data that encourages further exploration for testing the material either under conditions more appropriate to the intended point of use or even in practice. For example, bacterial inocula used in standardized testing (~10^5^–10^8^ CFU/mL) are significantly higher than those found in most potential end-use settings (e.g., ~10^2^–10^4^) [52]. 

Additionally, in order to validate the reproducibility of an antimicrobial material, ideally several different labs should perform the relevant standardised test method and achieve results that are all within the natural error range for such test [53]. Whilst the validity of individual test method data should be acknowledged, a growing need exists for precise and reproducible methods, as many different AMM´s fall short when being tested by independent reviewers [54]. 

In several cases, the test methods used to determine the efficacy of an AMM are inadequate due to a variety of factors including the incubation time/environmental conditions. In some cases this can artificially favour the increased and/or prolonged efficacy of the material, particularly by raising the humidity to >90%, a condition which is almost never seen in end-use environments [55,56]. Many of the materials used in AMM’s require moisture to be antimicrobial, metallic silver for example, which is ionised in the presence of moisture to form silver ions which have numerous antimicrobial properties. Therefore, knowledge of the length of time a surface remains moist for (the drying time of the deposit carrying the contaminating microorganism on the surface) is vital for an accurate judgement on the efficacy of the surface [57]. This reliance on moisture must be considered when testing surfaces for effectiveness at point of use. Indeed, knowing the time it takes for an inoculum (a suspension of microbial cells of any manner) applied as a liquid to dry in each environment is key to assessing the activity of many AMMs.

## 3. Standardised Tests for AMMs In Vitro

There are five general categories of test for an antimicrobial material in vitro: (1) high surface area to volume ratio tests, (2) agar zone of inhibition tests, (3) suspension tests, (4) adhesion tests, (5) biofilm tests [26]. These tests differ based on the mechanism of action they are intended to evaluate (among other factors) and are explored below. Many test methods are constructed and defined by several different test method development organisations such as ISO (International Standards Organisation), BS (British Standards), IBRG (International Biodeterioration Research Group), and ASTM (American Society for Testing and Materials). There have been many iterations and modifications of these methods described in the literature that deviate based on the individual preferences of the testing laboratory, which then makes comparison of data generated for similar materials in different laboratories problematic. 

### 3.1. Methods Constituting High Surface Area to Volume Ratio

**Figure 1:**
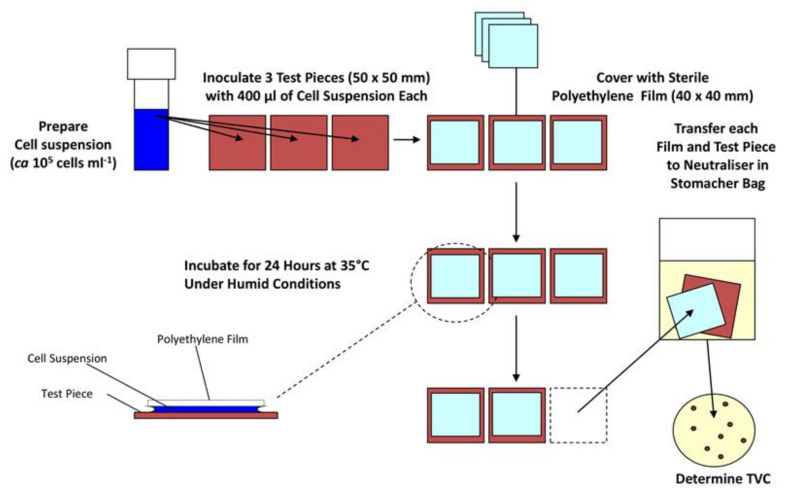
Diagram describing important steps in the ISO 22196 antimicrobial materials efficacy test.

These methods focus on maximising the contact between the surface and the microorganism, so the cells and the surface are essentially always touching and interacting. This is usually done by placing the bacteria between the test sample and another sterilised non-antimicrobial material such as glass or plastic. The most used test method for antimicrobial materials in this category is ISO 22196:2011 (and similar methods such as JIS Z 2801). Here, a surface is inoculated with a bacterial suspension of known concentration and volume (Figure 1). A polyethylene film is placed on top of the inoculum, and the material is incubated at 35 °C in upwards of 90% humidity for 24 h. Bacteria are removed from the surface by mechanical detachment and re-suspension in a neutralising diluent, before the number of colony forming units (CFUs) is determined by plate count [58]. This method is relatively straight-forward and cheap to run, and so has been widely adopted. However, none of the experimental conditions relate to end-use environments. Indeed, in the majority of cases, the opposite is true, ISO 22196:2011 keeps a microbial inoculum wet for the duration of a 24-h test, allowing the antimicrobial material to provide a sustained antimicrobial action by dissolving into the water and this way ensuring contact between the cell wall of a microorganism and the biocidal active substance—which will not mirror the conditions when an AMM is implemented. To overcome such shortcomings, recent developments include a test method where bacteria are aerosolised, so they are deposited onto a dry surface, in an attempt to reduce artificial antimicrobial action resulting from the deposition of a wet microbial inoculum [59]. However, dry deposition is not without its own challenges, for example ensuring reproducible inoculum quantity and ensuring a safe working environment from the release pathogens into the air.

Other methods using a high surface area to volume ratio have been reported in the literature. One method (a modification of ISO 22196:2011) involves inoculation of the antimicrobial material with a bacterial suspension within a film of agar (commonly made into a slurry) to ensure that contact is maintained between the AMM and the test organism. When recovering the bacteria from the material, a neutraliser is used to resuspend the inoculum (neutralising diluent is a key step in a majority of antimicrobial test methods to prevent superfluous interactions between the material and microorganism) and CFU counts are determined. However, it is possible that the slurry (when required) has a soiling effect on the material preventing it from performing its antimicrobial effect as efficiently as possible as different antimicrobial materials will present largely different diffusion rates and characteristics into an agar slurry [60,61]. 

Another modification of ISO 22196:2011 uses a filter, inoculated with bacteria, as the top layer rather than a polyethylene film perhaps to reduce loss of microorganisms through fewer manipulations (omitting drop inoculation) [62]. Alternatively, a liquid bacterial suspension can be sprayed (to simulate the typical deposition of airborne bacteria on to a surface by coughing, etc.) on to the antimicrobial material from a distance of 15cm, followed by live-dead staining after being air-dried for two minutes. Spraying of microbes rather than deposition has the advantage of being more representative of a patient in a hospital in most cases but is more complicated to run (and standardise) than simply placing a droplet of water on to the surface, as it requires specialist equipment.

In conclusion, several authors have developed methods that are related to ISO 22196, presumably due to their ease of use and relatively low cost. However, there are many limitations in the translation of results obtained to the point of use of the materials, with most relating to the dissimilarity of the conditions standardised in ISO 22196 compared to end-use environments, both clinical and non-clinical, and others arising from difficulties in comparing results from methods that slightly differ from laboratory to laboratory.

### 3.2. Agar Zone of Inhibition Methods

Methods that utilise zones of inhibition, for which there are two existing standardised methods, are relatively quick and simple but may provide only an indicator of whether any antimicrobial effect might be present under permanently wet conditions. The first, ISO 20645:2004 is based on a disk diffusion method, where the AMM is placed on top of an inoculated nutrient agar plate, incubated at the required temperature for a set time depending on the requirements for the bacteria being tested (such as at 37 °C for 24 h for *E. coli*) [63]. The second, AATCC 30 (which evaluates fungi rather than bacteria), places a spore suspension on to a solid agar medium and covers that with the AMM, inoculation with spores also occurs on top of the AMM after placement, the Petri dish is then sealed to maintain humidity and efficacy evaluation is based on macroscopic or microscopic visibility of fungi [64]. These methods have limitations such as (i) the incubation temperature is not relevant to the end use (often it is the optimal growth temperature for the test organism), (ii) incubation time is not reflective of expected cleaning protocols (FTS’s are likely to be touched more than once per day), and (iii) the nutrients present from the agar would likely not be present so abundantly on surfaces, all of which reduce the similarity to end-use environments. In addition, if the active substance is not emitted from the AMM, activity is unlikely to be measured, limiting applicability to a subset of AMMs. Furthermore, the viability of the test microorganisms may be compromised by the material being placed directly on top, even when exhibiting no antimicrobial effect. Nonetheless, the results provided from these test methods can allow a quick and simple approach to determining whether a material has at least some antimicrobial activity, which may be all that is required at the first stages of product development. Additionally, these methods can be used as a reliable quality control step during the production of an AMM once approved [65]. 

### 3.3. Suspension Methods

Suspension methods focus on inoculating and incubating bacteria in a liquid medium containing the antimicrobial surface, and then determining the remaining viable CFUs by taking an aliquot of this liquid and performing a dilution plate count using it. This allows assessment of materials that exhibit antimicrobial-release properties. However, due to the inoculum not being placed directly on the material, only surfaces that release antimicrobials can be tested. There are two current standardised methods. The first, ASTM E2149-13a, requires that the material is immersed in the medium (that is most appropriate for the bacteria used) following bacterial inoculation, then after incubation while shaking to enable increased contact of AMM to the bacteria, the CFU count is determined [66]. This method was developed with the intention of determining the effectiveness of silane QACs by agitating the suspension with sufficient vigour to cause cells to come in to contact with the QAC ‘tails’ [67], although this method is somewhat disputed. A modification to this method has also been developed whereby the entire suspension container is treated with the antimicrobial to prevent biofilms forming and to avoid complications arising from the extensive agitation that is required for the standard [67]. The second, JIS L 1902 (and also the absorption method within ISO 20743), describes the incubation of a porous material absorbing a specified volume of the appropriate medium, bacteria are then detached from the porous material using a stomacher in 20 mL neutralising diluent, and CFUs are determined in the resultant suspension [68]. For methods where an antimicrobial active substance has likely leached into liquid media (which will then be diluted and plated onto agar), which both of these suspension methods utilise, a neutralising solution is essential—to ensure continued antimicrobial action does not occur during the dilution and CFU determining incubation stages (24 h at the most appropriate temperature for the bacterial strain used). As these methods do not assess contact-killing materials, it is not possible to assess efficacy for using such material in some end-use conditions, for example, as a touch surface.

### 3.4. Adhesion Methods

These tests focus on quantifying the number of bacteria that can adhere to an antimicrobial material. There are two approaches to this form of testing. The first requires that the test surface is inoculated with the bacteria and incubated for 1 to 4 h. Non-adhering bacteria are removed and either the surface with attached cells is added to a liquid medium or an agar slab is placed on top of the surface and incubated (for counting CFUs), or the microorganisms can be stained (e.g., live-dead staining) to determine cells per unit area [69]. The second method specifies a flow of bacterial suspension through a chamber containing the antimicrobial material, then live-dead staining is performed on the cells attached to the surface to determine survival status of adhered cells. Alternatively, the cells are detached from the surface of the material and re-suspended so that the number of CFUs of the resultant suspension can be calculated [70]. Finally, a proliferation assay can be used, which involves inoculating an AMM for a given time, followed by rinsing and placing in a soy medium. The efficacy of the AMM is determined by the number of clonal counterparts produced by the surviving bacteria that are attached to the AMM, real-time spectrophotometer readings are essential to creating a growth curve to compare a control to the AMM [71]. In both methods, there are issues that arise from how effectively the detachment of bacteria occurs, even by sonication (which can cause the diluent to heat up), as this may also reduce the viability of the organisms and their growth on media.

### 3.5. Biofilm Methods

A biofilm is an assemblage of microorganisms that are associated with a surface and often become encased in a matrix of polysaccharide material [72,73,74]. They are one of the most common forms that microorganisms take on Earth and can cause significant problems when they form in certain artificial environments. Due to their impact on health, industry and the environment, the ability to either destroy a biofilm or to stop it from forming in the first instance using AMMs is a focus of increasing importance. However, biofilm development and testing are complex, and whilst recent efforts to advance standardised biofilm growth (e.g., ASTM E2647—20 and ASTM E3161—18) and efficacy testing of disinfectants against biofilm exist [75,76], methods for efficacy testing of anti-biofilm materials are limited and suffer from problems regarding quantification of the bacteria, as staining will offer little insight and sonication may cause a reduction in viability, although some use of bioreactors for biofilm testing is taking place e.g. [77].

## 4. Incubation/Environmental Factors Affecting the Efficacy of Antimicrobial Test Methods

If a test method should inform on the efficacy of a surface under end-use conditions, the environmental conditions of that test need to be considered carefully. For example, if moisture is essential for activity of an antimicrobial material, a test method that includes a high humidity, no airflow and warm temperature will result in the bacterial inoculum remaining wet for the duration of the test and will provide optimal results for that AMM. However, if that putative AMM would be used in a more realistic setting (such as a hospital ward), humidity is likely to be considerably lower (30% to 65%) as will the temperature (18 °C to 28 °C), and there will be air circulating (e.g., via movement of doors, people and air conditioning) [78]. Contaminating droplets of liquid are likely to dry quickly, reducing the time for which the AMM is active. Most existing standard methods for antimicrobial testing vary in their environmental condition stipulations, but only to a relatively small degree (as seen in Table 1). Environmental conditions such as these can alter the efficacy of an AMM, and therefore careful consideration should be given when designing or interpreting data from an antimicrobial test method.

Humidity- Humidity is an important determinant for the drying time of liquid droplets [79], and therefore is most likely linked to AMM efficacy. In most cases, a reduction in the humidity of the AMM results in a lower antimicrobial efficacy because of the reduction in moisture at the surface through evaporation. For example, when assessing the activity of a copper alloy surface (with varying copper quantities in the alloy), incubating at 37 °C and 100% relative humidity (RH) provides a 4-log reduction in around 30 min for all alloys higher than 70% copper content. However, when the environmental conditions are more analogous to that of an indoor room, at approximately 20 °C and 40–50% RH, the time taken to achieve the same 4-log reduction of viable bacterial load is doubled to 60 min [80]. In addition, silver ions released from zeolite have demonstrated significant antimicrobial effect at >90% RH, but the same composition showed no significant antimicrobial effects at 24% RH and a temperature of 20 °C. Although neither RH used in this study can be considered typical, it highlights the importance of the role that humidity plays in either increasing or reducing the antimicrobial efficacy of an AMM [55]. Noyce, Michels and Keevil [56] suggests that silver ions exhibit significantly decreased antimicrobial efficacy when the humidity is reduced to around 20%, again emphasizing the requirement for humidity to be considered when performing antimicrobial test methods. Furthermore, Ronan et al. [81] have shown that desiccation resistance is also significantly affected by the relative humidity, and this has downstream effects on the microbial survival on a surface, whereby survival of microorganisms can be greater at lower (25±%) relative humidity’s compared to higher (95±%), this work also highlights that interactions between bacterial species also allow for greater survival on materials compared to pure cultures. Finally, this work uses aerosolisation to deposit bacteria on to the surfaces, alleviating the disadvantages that droplets at high humidity possess on the antimicrobial efficacy of a material.

Temperature- Temperature has been shown to affect the survival of microorganisms on a surface, e.g., [82], and has also been shown to significantly affect the release of antimicrobials from a material [83] and probably the method (or at least the speed) with which they interact with the target species. Additionally, temperature does significantly affect the drying time of water on non-porous surfaces [79]—and therefore potentially affects antimicrobial efficacy due to the resulting change in humidity that occurs. 

Airflow- There is limited research into the effect of airflow on drying time and/or antimicrobial efficacy of a material. However, drying time of a liquid droplet on a non-porous material has been shown to be slower in the absence of airflow [79]. In addition, the transfer of infectious aerosols in hospitals is well understood and the ventilation system has been associated with much of this concern [84]. Thus, airflow is perhaps an important environmental factor that has been significantly overlooked in the design of testing AMM—even more so than humidity and temperature as it has never been considered in a relevant standardised test method. 

Surface topography- In addition to the environmental conditions described above, the surface features such as hydrophobicity and surface roughness can have a significant impact on how long moisture stays on its surface [85]. While a hydrophilic surface allows water to spread out evenly over the whole surface and to dry evenly, a hydrophobic surface results in the formation of droplets that cover the surface only in parts and that will dry out slower compared to the same volume when place on a hydrophilic material. Hydrophobicity and surface roughness will dictate the contact angle created with a droplet; when the contact angle is larger from an increased hydrophobicity, the droplet will possess a lower surface area to volume ratio, also therefore decreasing drying time [86]. 

Spreading of the inoculum- Spreading plays an important part of some antimicrobial tests. However, increased care should be taken when considering the spreading of the inoculum, as inconsistencies in the spreading process are almost inevitable between laboratories and even individual persons, leading to differences in the drying time of the droplets/inoculums and the repeatability of the results. Spreading must also be considered alongside hydrophobicity data of the AMM, as hydrophobicity will partially dictate how easily, if at all, the inoculum spreads across the surface, further increasing the possibility of unreliable results [87]. If hydrophobicity/hydrophilicity is part of the function of the coating comparing results from materials on which the inoculum exhibits dissimilar spreading rates may be problematic and will need to be taken into consideration.

Inoculum density- Little work has been done linking the addition of an inoculum to the drying time of a droplet. However, it is likely to cause a difference to drying time either by interaction of the bacteria with the inoculum (to perform metabolic processes) and surface, or simply by taking up a certain volume of the inoculum. The impact of soiling agents in the inoculum may have effects not only on the drying rate, but also by having an impact on the susceptibility of the target species to the effects of desiccation.

Exposure time- A longer exposure time will allow more interactions between the microorganism and the surface, potentially causing an increase in antimicrobial effect (or, under extended conditions its recovery). All current test methods take this factor into account and specify an exact incubation period for the antimicrobial test to occur—however, relating this period of time to the end-use is essential, particularly where an AMM is anticipated to be touched with high frequency.

Threshold to achieve antimicrobial claim- Often a 2–3 log reduction in microbial viability is required although it is at the discretion of all involved parties to agree to a value that is considered to be appropriate to the end-use. The scale and speed of an effect must be appropriate to the benefit that is intended/required.

In addition to those described above, other influences that can have an effect on efficacy in real-life situations and are often not considered. For example, when testing include the use of cleaning agents on the AMM, the cleanliness of a surface over time and the aging of the AMM, especially when using structured surfaces, where topography can change over time.

## 5. Discussion

As described above, a variety of test methods exist that are capable of determining the antimicrobial activity of a material. With this comes variations and alterations to each method based on the specific conditions of the testing laboratory at the time and date of testing, among other factors, that will likely affect the accuracy and reliability of the resulting antimicrobial efficacy of the material. It is worth noting that some modifications may allow the method to be more appropriate to the question being asked. Whilst the methodological variation from a standard method is with good intention, for example, using a temperature the investigator considers closer to room temperature, the effect on reproducibility can be profound. For example, if two different labs were to consider the antimicrobial efficacy of a given material, following a standardised test method, but one used a test chamber/container that was bigger than the other lab, there would be an impact on the time it takes to reach the intended RH. This would affect the ability of the inoculum to remain wet on the surface, and therefore alter the time the antimicrobial material is likely to be active. The same can be true for other experimental factors such as the method of achieving the desired RH (using concentrations of saturated salts), controlling temperature (placing a chamber in an incubator compared to using a heat mat), the speed at which samples are removed from a chamber for different time points and so on. Often, these seemingly small changes to methodology alter the reproducibility between laboratories. For example, one study asked different labs to assess the antimicrobial efficacy of the same materials (polyamide 6 and an antibacterial zinc additive at multiple concentrations) using ISO 22196:2011. Following analysis of the data, several factors were found to be inconsistent between labs, such as extraction medium and method of cell enumeration, which led to a large disparity in the final results obtained, where the microbial reduction ranged from 1.73-log to 6.3-log for the same antimicrobial compound [88]. Care should also be taken to ensure the method of cell enumeration is not only consistent across all laboratories using a test method but also that it is appropriate for the specific test method that is being used, live-dead staining for instance is not always effective, particularly when assessing biofilms [89].

Whilst many of the test methods have been designed to be useable and achievable in a range of laboratories, issues remain in terms of the validity of the methods. Most critically, how can efficacy in realistic uses be inferred using the data that the test methods generate—because the current methods are not reflective of realistic in-use conditions. It is abundantly clear that a considerable improvement is required in test methodology of antimicrobial materials if subsequent results are used to support efficacy claims for different environmental conditions while in use. This clash of intention and inference would be best addressed by an interdisciplinary approach to new antimicrobial efficacy test method development. However, the process of making any changes to current test methods should be taken with due diligence, as unexpected consequences are possible. For example, temperature and drying time of a droplet have been found to affect the rate of detachment of *Bacillus* spores from a surface once the droplet has dried, which will be more likely to occur if humidity is lowered to 40–50% to keep in-line with a majority of indoor acclimatised settings [90]. 

Finally, there have been improvements in recent years that show promise in creating more realistic and higher reliability test methods. One potentially useful addition would be the addition of video protocols to work alongside traditional paper protocols, allowing users to get an exact understanding of the nuances related to the test method. Another answer may lie in the recent advances in both computer simulations of heat/mass transfer and in the decreasing cost of improved microfluidic devices that provide the potential for both cheap and reliable test methods [91], which when integrated in to an environmental control chamber could provide realistic and reliable antimicrobial efficacy assessment.

## 6. Conclusions

The utilisation and effectiveness of antimicrobial materials in an end-use scenario is likely considerably different from results achieved from the testing methods currently in use and standardised. Using test methods that better reflect end-user environments would enable more realistic efficacy assessment. An approach to efficacy assessment of antimicrobial materials should build on current standard methods but seek to understand and design methods that model the behaviour of the antimicrobial material as if it were placed in its intended end-use setting, such as by adding additional environmental conditions. However, the increased difficulty and cost, and lack of models that explain the impact of drying time of liquids deposited on a surface will require significant work to fully understand and develop into new AMM efficacy test methods.

## Figures and Tables

**Table 1:** Examples of methodological test conditions used in several standardized efficacy test methods.

Name of Standard	Material Type	Organisms Used	Time	Humidity	Temperature (°C)	Criteria for Being AM	Other Conditions
EN ISO 846:1997	Plastics	*A. niger*; *P. funiculosum*; *P. variotii*; *G. virens*; *C. globosum*; *P. aeruginosa*	4 weeks	97%	29	No visible growth to the naked eye	None
EN ISO 20743:2013	Textiles	*S. aureus*; *K. pneumoniae*	18–24 h	70%	37±2	CFUs < 1 × 10^5 reduction of life of 50%	None
EN ISO 22196:2011	Plastics/non-porous	*S. aureus*; *E. coli*	24+/-1 h	>90%	35±2	Agreed upon by case	None
ISO 27447:2019	Ceramics/photocatalytic	*S. aureus*; *K. pneumoniae; E. coli*	18–24 h	No mention	37±1	Log reduction of 0.8	None
EN 16615:2015	Non-porous surfaces	*S. aureus*; *E. hirae*; *P. aeruginosa; C. albicans*	60 min	No mention	4–30±2	5 log reduction	None
ISO 18184:2019	Textiles	Influenza A; Feline Calcivirus	3–5 days	No mention	34	Antiviral efficiency of >2	5% CO_2_
ISO 21702:2019	Plastics/non-porous	Influenza A; Feline Calcivirus	2–3 days	No mention	34	Agreed upon by case	5% CO_2_
ISO 18061:2014	Ceramics/photocatalytic	Bacteriophage Q beta; *E. coli*	24 h	No mention	37±1	Log reduction of 0.8	None

## References

[1] Mirhoseini S.H., Nikaeen M., Khanahmad H., Hatamzadeh M., Hassanzadeh A. (2015). Monitoring of airborne bacteria and aerosols in different wards of hospitals-Particle counting usefulness in investigation of airborne bacteria. Ann. Agric. Environ. Med..

[2] Jung I., Choi W., Kim J., Wang E., Park S.-W., Lee W.-J., Choi J., Kim H., Uh Y., Kim Y. (2019). Nosocomial person-to-person transmission of severe fever with thrombocytopenia syndrome. Clin. Microbiol. Infect..

[3] Stephens B., Azimi P., Thoemmes M.S., Heidarinejad M., Allen J.G., Gilbert J.A. (2019). Microbial exchange via fomites and implications for human health. Curr. Pollut. Rep..

[4] Pommepuy M., Butin M., Derrien A., Gourmelon M., Colwell R., Cormier M. (1996). Retention of enteropathogenicity by viable but nonculturable Escherichia coli exposed to seawater and sunlight. Appl. Environ. Microbiol..

[5] Neely A.N. (2000). A survey of gram-negative bacteria survival on hospital fabrics and plastics. J. Burn Care Rehabil..

[6] Heller L.C., Edelblute C.M. (2018). Long-term metabolic persistence of gram-positive bacteria on health care-relevant plastic. Am. J. Infect. Control.

[7] Jaradat Z.W., Ababneh Q.O., Sha’aban S.T., Alkofahi A.A., Assaleh D., Al Shara A. (2020). Methicillin Resistant Staphylococcus aureus and public fomites: A review. Pathog. Glob. Health.

[8] Xiao S., Jones R.M., Zhao P., Li Y. (2019). The dynamic fomite transmission of Methicillin-resistant Staphylococcus aureus in hospitals and the possible improved intervention methods. Build. Environ..

[9] Kraay A.N., Hayashi M.A., Hernandez-Ceron N., Spicknall I.H., Eisenberg M.C., Meza R., Eisenberg J.N. (2018). Fomite-mediated transmission as a sufficient pathway: A comparative analysis across three viral pathogens. BMC Infect. Dis..

[10] Weber K.L., LeSassier D.S., Kappell A.D., Schulte K.Q., Westfall N., Albright N.C., Godbold G.D., Palsikar V., Acevedo C.A., Ternus K.L. (2020). Simulating transmission of ESKAPE pathogens plus C. difficile in relevant clinical scenarios. BMC Infect. Dis..

[11] Suwantarat N., Supple L.A., Cadnum J.L., Sankar T., Donskey C.J. (2017). Quantitative assessment of interactions between hospitalized patients and portable medical equipment and other fomites. Am. J. Infect. Control..

[12] Huslage K., Rutala W.A., Sickbert-Bennett E., Weber D.J. (2010). A quantitative approach to defining “high-touch” surfaces in hospitals. Infect. Control. Hosp. Epidemiol..

[13] Cheng V., Chau P., Lee W., Ho S., Lee D., So S., Wong S., Tai J., Yuen K. (2015). Hand-touch contact assessment of high-touch and mutual-touch surfaces among healthcare workers, patients, and visitors. J. Hosp. Infect..

[14] Liu X., Chang Y.-C. (2020). An emergency responding mechanism for cruise epidemic prevention—taking COVID-19 as an example. Mar. Policy.

[15] Meinke R., Meyer B., Frei R., Passweg J., Widmer A.F. (2012). Equal efficacy of glucoprotamin and an aldehyde product for environmental disinfection in a hematologic transplant unit: A prospective crossover trial. Infect. Control. Hosp. Epidemiol..

[16] Jennings M.C., Minbiole K.P., Wuest W.M. (2015). Quaternary ammonium compounds: An antimicrobial mainstay and platform for innovation to address bacterial resistance. ACS Infect. Dis..

[17] Han Y., Zhou Z.-C., Zhu L., Wei Y.-Y., Feng W.-Q., Xu L., Liu Y., Lin Z.-J., Shuai X.-Y., Zhang Z.-J. (2019). The impact and mechanism of quaternary ammonium compounds on the transmission of antibiotic resistance genes. Environ. Sci. Pollut. Res..

[18] Hosein I., Madeloso R., Nagaratnam W., Villamaria F., Stock E., Jinadatha C. (2016). Evaluation of a pulsed xenon ultraviolet light device for isolation room disinfection in a United Kingdom hospital. Am. J. Infect. Control.

[19] Page K., Wilson M., Parkin I.P. (2009). Antimicrobial surfaces and their potential in reducing the role of the inanimate environment in the incidence of hospital-acquired infections. J. Mater. Chem..

[20] Dunne S.S., Ahonen M., Modic M., Crijns F.R., Keinänen-Toivola M.M., Meinke R., Keevil C.W., Gray J., O’Connell N.H., Dunne C.P. (2018). Specialized cleaning associated with antimicrobial coatings for reduction of hospital-acquired infection: Opinion of the COST Action Network AMiCI (CA15114). J. Hosp. Infect..

[21] Dauvergne E., Mullié C. (2021). Brass Alloys: Copper-Bottomed Solutions against Hospital-Acquired Infections?. Antibiotics.

[22] Salazar H., Martins P., Santos B., Fernandes M., Reizabal A., Sebastián V., Botelho G., Tavares C.J., Vilas-Vilela J.L., Lanceros-Mendez S. (2020). Photocatalytic and antimicrobial multifunctional nanocomposite membranes for emerging pollutants water treatment applications. Chemosphere.

[23] Lemire J.A., Harrison J.J., Turner R.J. (2013). Antimicrobial activity of metals: Mechanisms, molecular targets and applications. Nat. Rev. Microbiol..

[24] Oosterhof J.J., Buijssen K.J., Busscher H.J., van der Laan B.F., van der Mei H.C. (2006). Effects of quaternary ammonium silane coatings on mixed fungal and bacterial biofilms on tracheoesophageal shunt prostheses. Appl. Environ. Microbiol..

[25] Khadka P., Haque M., Krishnamurthi V.R., Niyonshuti I., Chen J., Wang Y. (2018). Quantitative Investigations Reveal New Antimicrobial Mechanism of Silver Nanoparticles and Ions. Biophys. J..

[26] Sjollema J., Zaat S.A.J., Fontaine V., Ramstedt M., Luginbuehl R., Thevissen K., Li J., van der Mei H.C., Busscher H.J. (2018). In vitro methods for the evaluation of antimicrobial surface designs. Acta Biomater.

[27] Kaur R., Liu S. (2016). Antibacterial surface design–Contact kill. Prog. Surf. Sci..

[28] Shukla A., Fleming K.E., Chuang H.F., Chau T.M., Loose C.R., Stephanopoulos G.N., Hammond P.T. (2010). Controlling the release of peptide antimicrobial agents from surfaces. Biomaterials.

[29] Wu P., Grainger D.W. (2006). Drug/device combinations for local drug therapies and infection prophylaxis. Biomaterials.

[30] Buhat C.A.H., Lutero D.S., Olave Y.H., Torres M.C., Rabajante J.F. (2020). Modeling the Transmission of Respiratory Infectious Diseases in Mass Transportation Systems. medRxiv.

[31] Hans M., Erbe A., Mathews S., Chen Y., Solioz M., Mücklich F. (2013). Role of copper oxides in contact killing of bacteria. Langmuir.

[32] Tiller J.C., Liao C.-J., Lewis K., Klibanov A.M. (2001). Designing surfaces that kill bacteria on contact. Proc. Natl. Acad. Sci. USA.

[33] Campoccia D., Montanaro L., Arciola C.R. (2013). A review of the biomaterials technologies for infection-resistant surfaces. Biomaterials.

[34] Yan X., He B., Liu L., Qu G., Shi J., Hu L., Jiang G. (2018). Antibacterial mechanism of silver nanoparticles in Pseudomonas aeruginosa: Proteomics approach. Metallomics.

[35] Sondi I., Salopek-Sondi B. (2004). Silver nanoparticles as antimicrobial agent: A case study on E. coli as a model for Gram-negative bacteria. J. Colloid Interface Sci..

[36] Warnes S., Keevil C. (2011). Mechanism of copper surface toxicity in vancomycin-resistant enterococci following wet or dry surface contact. Appl. Environ. Microbiol..

[37] Chandraleka S., Ramya K., Chandramohan G., Dhanasekaran D., Priyadharshini A., Panneerselvam A. (2014). Antimicrobial mechanism of copper (II) 1, 10-phenanthroline and 2, 2′-bipyridyl complex on bacterial and fungal pathogens. J. Saudi Chem. Soc..

[38] Vincent M., Duval R.E., Hartemann P., Engels-Deutsch M. (2018). Contact killing and antimicrobial properties of copper. J. Appl. Microbiol..

[39] Giannousi K., Lafazanis K., Arvanitidis J., Pantazaki A., Dendrinou-Samara C. (2014). Hydrothermal synthesis of copper based nanoparticles: Antimicrobial screening and interaction with DNA. J. Inorg. Biochem..

[40] Fakhri A., Behrouz S. (2015). Assessment of SnS2 nanoparticles properties for photocatalytic and antibacterial applications. Sol. Energy.

[41] Vaishampayan A., de Jong A., Wight D.J., Kok J., Grohmann E. (2018). A novel antimicrobial coating represses biofilm and virulence-related genes in methicillin-resistant Staphylococcus aureus. Front. Microbiol..

[42] Bolhuis A., Hand L., Marshall J.E., Richards A.D., Rodger A., Aldrich-Wright J. (2011). Antimicrobial activity of ruthenium-based intercalators. Eur. J. Pharm. Sci..

[43] Chen F., Yang X., Wu Q. (2009). Antifungal capability of TiO2 coated film on moist wood. Build. Environ..

[44] Sichel C., Tello J., De Cara M., Fernández-Ibáñez P. (2007). Effect of UV solar intensity and dose on the photocatalytic disinfection of bacteria and fungi. Catal. Today.

[45] Lee H.U., Lee S.C., Choi S., Son B., Lee S.M., Kim H.J., Lee J. (2013). Efficient visible-light induced photocatalysis on nanoporous nitrogen-doped titanium dioxide catalysts. Chem. Eng. J..

[46] Burda C., Lou Y., Chen X., Samia A.C., Stout J., Gole J.L. (2003). Enhanced nitrogen doping in TiO2 nanoparticles. Nano Lett..

[47] Akhavan O., Ghaderi E. (2010). Toxicity of graphene and graphene oxide nanowalls against bacteria. ACS Nano.

[48] Azizi-Lalabadi M., Hashemi H., Feng J., Jafari S.M. (2020). Carbon nanomaterials against pathogens; the antimicrobial activity of carbon nanotubes, graphene/graphene oxide, fullerenes, and their nanocomposites. Adv. Colloid Interface Sci..

[49] Zheng H., Ji Z., Roy K.R., Gao M., Pan Y., Cai X., Wang L., Li W., Chang C.H., Kaweeteerawat C. (2019). Engineered graphene oxide nanocomposite capable of preventing the evolution of antimicrobial resistance. ACS Nano.

[50] Holmes P., Currie E., Thies J., Van der Mei H., Busscher H., Norde W. (2009). Surface-modified nanoparticles as a new, versatile, and mechanically robust nonadhesive coating: Suppression of protein adsorption and bacterial adhesion. J. Biomed. Mater. Res. Part A: An. Off. J. Soc. Biomater. Jpn. Soc. Biomater. Aust. Soc. Biomater. Korean Soc. Biomater..

[51] Salwiczek M., Qu Y., Gardiner J., Strugnell R.A., Lithgow T., McLean K.M., Thissen H. (2014). Emerging rules for effective antimicrobial coatings. Trends Biotechnol..

[52] Sinclair R.G., Gerba C.P. (2011). Microbial contamination in kitchens and bathrooms of rural cambodian village households. Lett. Appl. Microbiol..

[53] Bloomfield S.F., Looney E. (1992). Evaluation of the repeatability and reproducibility of European suspension test methods for antimicrobial activity of disinfectants and antiseptics. J. Appl. Bacteriol..

[54] Ioannidis J.P. (2005). Why most published research findings are false. PLoS Med..

[55] Michels H., Noyce J., Keevil C.W. (2009). Effects of temperature and humidity on the efficacy of methicillin-resistant Staphylococcus aureus challenged antimicrobial materials containing silver and copper. Lett. Appl. Microbiol..

[56] Noyce J., Michels H., Keevil C. (2006). Potential use of copper surfaces to reduce survival of epidemic meticillin-resistant Staphylococcus aureus in the healthcare environment. J. Hosp. Infect..

[57] Rai M., Yadav A., Gade A. (2009). Silver nanoparticles as a new generation of antimicrobials. Biotechnol. Adv..

[58] International Organization for Standardization, ISO (2011). 22196: Measurement of antibacterial activity on plastics and other non-porous surfaces. International Organization for Standardization: Geneva, Switzerland. https://www.iso.org/standard/54431.html.

[59] McDonald M., Wesgate R., Rubiano M., Holah J., Denyer S.P., Jermann C., Maillard J.Y. (2020). Impact of a dry inoculum deposition on the efficacy of copper-based antimicrobial surfaces. J. Hosp. Infect..

[60] Sjollema J., Keul H., van der Mei H., Dijkstra R., Rustema-Abbing M., de Vries J., Loontjens T., Dirks T., Busscher H. (2017). A Trifunctional, Modular Biomaterial Coating: Nonadhesive to Bacteria, Chlorhexidine-Releasing and Tissue-Integrating. Macromol. Biosci..

[61] European Chemicals Agency (2016). Guidance on the Biocidal Products Regulation.

[62] Van de Lagemaat M., Grotenhuis A., van de Belt-Gritter B., Roest S., Loontjens T.J., Busscher H.J., van der Mei H.C., Ren Y. (2017). Comparison of methods to evaluate bacterial contact-killing materials. Acta Biomater..

[63] International Organization for Standardization, ISO (2004). 20645. Textile fabrics — Determination of antibacterial activity — Agar diffusion plate test, International Organization for Standardization: Geneva, Switzerland. https://www.iso.org/standard/35499.html.

[64] American Association of Textile Chemists and Colorists (2017). Antifungal Activity, Assessment on Textile Materials: Mildew and Rot Resistance of Textile Materials. American Association of Textile Chemists and Colorists: Research Triangle Park, NC, USA. https://members.aatcc.org/store/tm30/491/.

[65] Åhman J., Matuschek E., Kahlmeter G. (2019). The quality of antimicrobial discs from nine manufacturers—EUCAST evaluations in 2014 and 2017. Clin. Microbiol. Infect..

[66] ASTM International, ASTM (2013). E2149-13A. Standard Test Method for Determining the Antimicrobial Activity of Antimicrobial Agents Under Dynamic Contact Conditions.

[67] Caschera A., Mistry K.B., Bedard J., Ronan E., Syed M.A., Khan A.U., Lough A.J., Wolfaardt G., Foucher D.A. (2019). Surface-attached sulfonamide containing quaternary ammonium antimicrobials for textiles and plastics. RSC Adv..

[68] International Organization for Standardization, ISO (2013). 20743-Textiles-Determination of Antibacterial Activity of Textile Products. International Organization for Standardization: Geneva, Switzerland. https://www.iso.org/standard/59586.html.

[69] Héquet A., Humblot V., Berjeaud J.-M., Pradier C.-M. (2011). Optimized grafting of antimicrobial peptides on stainless steel surface and biofilm resistance tests. Colloids Surf. B Biointerfaces.

[70] Albright V., Zhuk I., Wang Y., Selin V., van de Belt-Gritter B., Busscher H.J., van der Mei H.C., Sukhishvili S.A. (2017). Self-defensive antibiotic-loaded layer-by-layer coatings: Imaging of localized bacterial acidification and pH-triggering of antibiotic release. Acta Biomater..

[71] Alt V., Bechert T., Steinrücke P., Wagener M., Seidel P., Dingeldein E., Domann E., Schnettler R. (2004). In vitro testing of antimicrobial activity of bone cement. Antimicrob. Agents Chemother..

[72] Schachter B. (2003). Slimy business—the biotechnology of biofilms. Nat. Biotechnol..

[73] Kim H.-S., Lee J.Y., Ham S.-Y., Lee J.H., Park J.-H., Park H.-D. (2019). Effect of biofilm inhibitor on biofouling resistance in RO processes. Fuel.

[74] Donlan R.M. (2002). Biofilms: Microbial life on surfaces. Emerg. Infect. Dis..

[75] Goeres D.M., Walker D.K., Buckingham-Meyer K., Lorenz L., Summers J., Fritz B., Goveia D., Dickerman G., Schultz J., Parker A.E. (2019). Development, standardization, and validation of a biofilm efficacy test: The single tube method. J. Microbiol. Methods.

[76] ASTM (2019). E2871-19, Standard Test Method for Determining Disinfectant Efficacy Against Biofilm Grown in the CDC Biofilm Reactor Using the Single Tube Method.

[77] Dumitrache A., Eberl H.J., Allen D.G., Wolfaardt G.M. (2015). Mathematical modeling to validate on-line co2 measurements as a metric for cellulolytic biofilm activity in continuous-flow bioreactors. Biochem. Eng. J..

[78] Department of Health (2007). HTM 03-01: Specialised Ventilation for Healthcare Premises: Part A—Design and Validation.

[79] Redfern J., Tucker J., Simmons L.M., Askew P., Stephan I., Verran J. (2018). Environmental and Experimental Factors Affecting Efficacy Testing of Nonporous Plastic Antimicrobial Surfaces. Methods Protoc..

[80] Ojeil M., Jermann C., Holah J., Denyer S.P., Maillard J.-Y. (2013). Evaluation of new in vitro efficacy test for antimicrobial surface activity reflecting UK hospital conditions. J. Hosp. Infect..

[81] Ronan E., Yeung C.W., Hausner M., Wolfaardt G.M. (2013). Interspecies interaction extends bacterial survival at solid–air interfaces. Biofouling.

[82] Redfern J., Verran J. (2017). Effect of humidity and temperature on the survival of Listeria monocytogenes on surfaces. Lett. Appl. Microbiol..

[83] Kashiri M., Cerisuelo J.P., Domínguez I., López-Carballo G., Hernández-Muñoz P., Gavara R. (2016). Novel antimicrobial zein film for controlled release of lauroyl arginate (LAE). Food Hydrocoll..

[84] Tang J., Li Y., Eames I., Chan P., Ridgway G. (2006). Factors involved in the aerosol transmission of infection and control of ventilation in healthcare premises. J. Hosp. Infect..

[85] Richard E., Dubois T., Allion-Maurer A., Jha P.-K., Faille C. (2020). Hydrophobicity of abiotic surfaces governs droplets deposition and evaporation patterns. Food Microbiol..

[86] Kulinich S., Farzaneh M. (2009). Effect of contact angle hysteresis on water droplet evaporation from super-hydrophobic surfaces. Appl. Surf. Sci..

[87] Nakajima A. (2011). Design of hydrophobic surfaces for liquid droplet control. NPG Asia Mater..

[88] Wiegand C., Völpel A., Ewald A., Remesch M., Kuever J., Bauer J., Griesheim S., Hauser C., Thielmann J., Tonndorf-Martini S. (2018). Critical physiological factors influencing the outcome of antimicrobial testing according to ISO 22196/JIS Z 2801. PLoS ONE.

[89] Netuschil L., Auschill T.M., Sculean A., Arweiler N.B. (2014). Confusion over live/dead stainings for the detection of vital microorganisms in oral biofilms—Which stain is suitable?. BMC Oral Health.

[90] Faille C., Bihi I., Ronse A., Ronse G., Baudoin M., Zoueshtiagh F. (2016). Increased resistance to detachment of adherent microspheres and Bacillus spores subjected to a drying step. Colloids Surf. B Biointerfaces.

[91] Sun H., Chan C.-W., Wang Y., Yao X., Mu X., Lu X., Zhou J., Cai Z., Ren K. (2019). Reliable and reusable whole polypropylene plastic microfluidic devices for a rapid, low-cost antimicrobial susceptibility test. Lab. A Chip.

